# *CD209* Genetic Polymorphism and Tuberculosis Disease

**DOI:** 10.1371/journal.pone.0001388

**Published:** 2008-01-02

**Authors:** Fredrik O. Vannberg, Stephen J. Chapman, Chiea C. Khor, Kerrie Tosh, Sian Floyd, Dolly Jackson-Sillah, Amelia Crampin, Lifted Sichali, Boubacar Bah, Per Gustafson, Peter Aaby, Keith P. W. J. McAdam, Oumou Bah-Sow, Christian Lienhardt, Giorgio Sirugo, Paul Fine, Adrian V. S. Hill

**Affiliations:** 1 Wellcome Trust Centre for Human Genetics, University of Oxford, Oxford, United Kingdom; 2 London School of Hygiene and Tropical Medicine, London, United Kingdom; 3 Medical Research Council Laboratories, Fajara, The Gambia; 4 Karonga Prevention Study, Chilumba, Malawi; 5 University Ignace Deen, Conakry, Republic of Guinea; 6 Bandim Health Project, Bissau, Guinea-Bissau; 7 Institut de Recherche pour le Developpement (IRD), UMR 145, Dakar, Senegal; University of Stellenbosch, South Africa

## Abstract

**Background:**

Tuberculosis causes significant morbidity and mortality worldwide, especially in sub-Saharan Africa. DC-SIGN, encoded by *CD209*, is a receptor capable of binding and internalizing *Mycobacterium tuberculosis*. Previous studies have reported that the *CD209* promoter single nucleotide polymorphism (SNP)-336A/G exerts an effect on *CD209* expression and is associated with human susceptibility to dengue, HIV-1 and tuberculosis in humans. The present study investigates the role of the *CD209* -336A/G variant in susceptibility to tuberculosis in a large sample of individuals from sub-Saharan Africa.

**Methods and Findings:**

A total of 2,176 individuals enrolled in tuberculosis case-control studies from four sub-Saharan Africa countries were genotyped for the *CD209* -336A/G SNP (rs4804803). Significant overall protection against pulmonary tuberculosis was observed with the -336G allele when the study groups were combined (n = 914 controls vs. 1262 cases, Mantel-Haenszel 2x2 χ^2^ = 7.47, *P* = 0.006, odds ratio = 0.86, 95%CI 0.77–0.96). In addition, the patients with -336GG were associated with a decreased risk of cavitory tuberculosis, a severe form of tuberculosis disease (n = 557, Pearson's 2×2 χ^2^ = 17.34, *P* = 0.00003, odds ratio = 0.42, 95%CI 0.27–0.65). This direction of association is opposite to a previously observed result in a smaller study of susceptibility to tuberculosis in a South African Coloured population, but entirely in keeping with the previously observed protective effect of the -336G allele.

**Conclusion:**

This study finds that the *CD209* -336G variant allele is associated with significant protection against tuberculosis in individuals from sub-Saharan Africa and, furthermore, cases with -336GG were significantly less likely to develop tuberculosis-induced lung cavitation. Previous *in vitro* work demonstrated that the promoter variant -336G allele causes down-regulation of *CD209* mRNA expression. Our present work suggests that decreased levels of the DC-SIGN receptor may therefore be protective against both clinical tuberculosis in general and cavitory tuberculosis disease in particular. This is consistent with evidence that Mycobacteria can utilize DC-SIGN binding to suppress the protective pro-inflammatory immune response.

## Introduction

*Mycobacterium tuberculosis* infection causes significant morbidity and mortality throughout the world, particularly in resource-poor countries. *M. tuberculosis* infects one third of the human population and approximately two million persons die of tuberculosis every year [1]. Over the next 20 years the WHO estimates that another billion people will become newly infected with *M. tuberculosis* and more than 25 million will die from the disease, despite the availability of antibiotics [2], [3]. In sub-Saharan Africa, the rates of tuberculosis range from 50 to greater than 300 per 100,000 individuals [2]. It is now well-established that there is a host genetic component to susceptibility to tuberculosis [4]. Evidence to support this includes the observation of familial clustering of disease with higher concordance of tuberculosis disease in monozygotic versus dizygotic twins [5], the ethnic clustering of tuberculosis disease with a higher prevalence of tuberculosis in individuals of recent African descent [6], as well as the demonstration of both common polymorphisms and rare mutations which confer susceptibility to mycobacterial species in humans [7].

Dendritic Cell-Specific ICAM3-Grabbing Non-integrin (DC-SIGN), encoded by *CD209* on chromosome 19p13.3, is a C-type lectin that is expressed on subsets of dendritic cells (DCs) and alveolar macrophages [8]–[10]. DC-SIGN has been demonstrated to bind a variety of ligands [11]; endogenous ligands include endothelial cells through ICAM-2, T-lymphocytes through ICAM-3, neutrophils through MAC-1 and various endogenous glycosylated structures [11]–[15]; exogenous ligands include glycosylated moieties on *M. tuberculosis*, *M. leprae*, *Bacillus Calmette-Guérin* (BCG), *Heliobacter pylori*, *Streptococcus pneumoniae*, *Klebsiella pneumoniae*, HIV-1, HIV-2, SIV-1, Dengue virus, Ebola Virus, Cytomegalovirus, Hepatitis C virus, *Schistosoma mansoni*, *Leishmania pifanoi* and *Candida albicans* [10], [16]–[28] (and reviewed in [29]).

DC-SIGN is a major receptor for *M. tuberculosis* on immune cell subsets and has been shown to bind mannosylated lipoarabomannan (ManLAM) present on virulent, but not avirulent, strains of *Mycobacterium spp.* [13]. This binding can lead to internalization of *M. tuberculosis* and infection of DCs and alveolar macrophages [22]. DC-SIGN may also have additional roles in suppressing Toll-like receptor (TLR) signaling, thereby influencing Th1/Th2 balance (proinflammatory/anti-inflammatory) and causing *M. tuberculosis*-induced immune suppression [22], [29]. This effect is mediated by ManLAM and increased secretion of ManLAM from Mycobacteria may be crucial to tuberculosis disease progression [29].

There has been considerable recent interest in the role of *CD209* variation in human susceptibility to infectious diseases including *M. tuberculosis* and *M. leprae,* HIV-1, and Dengue [18], [30]–[32]. All but the *M. leprae* case-control study found an association of the CD209 -336A/G promoter SNP with infectious disease susceptibility or protection. Martin and colleagues demonstrated that the -336G allele was associated with susceptibility to parenteral but not mucosal HIV-1 infection, although this was not replicated in individuals of recent African descent [31]. Sakuntabhai and colleagues found that the -336G allele was protective against Dengue fever versus Dengue hemorrhagic fever, but not against controls [32]. Barreiro and colleagues found an association between with the -871G and -336A alleles and protection against tuberculosis in South African Cape Coloureds, but not against leprosy in Pakistan [18], [30]. Although the *CD209* -871 SNP was found to be the polymorphism most significantly associated with tuberculosis susceptibility in the South African Cape Coloureds, Barreiro and colleagues found that this SNP is not polymorphic in other African countries. The relatively high prevalence of the -871 SNP in the South African Cape Coloureds cases and controls is due to their inherent admixture with European and Asian populations.

Sakuntabhai and colleagues also presented evidence that the *CD209* -336G allele disrupts a potential SP-1-like transcription factor binding site and that this allele is associated with lower *CD209* expression *in vitro* than the ancestral -336A variant [32]. On the basis of the importance of DC-SIGN in *M. tuberculosis* binding and signaling events, and in an attempt to replicate the findings of Barreiro and colleagues in a larger population, we genotyped the *CD209* -336A/G SNP in five tuberculosis case-control studies from four different sub-Saharan African countries.

## Methods

### Sample Information

The tuberculosis case control studies from The Gambia (A), Republic of Guinea and Guinea Bissau [33], The Gambia (B) [34], and Malawi [35] have been previously described in detail. Briefly, tuberculosis case definitions for all studies depended upon confirmation of *M. tuberculosis* positivity by culture, smear, or histology. HIV-1 testing was routinely performed and the percentage of HIV-1 positive individuals among the tuberculosis index cases and controls for the various studies are as follows: The Gambia (A), cases 7.5%, controls 5.2%; Republic of Guinea, cases 9.0%, controls 5.1%; Guinea Bissau, cases 23.8%, controls 16.2%; Malawi, cases 62.2%, controls 33.5%. The Gambia (B) cohort has 330 confirmed HIV-1 negative tuberculosis cases, with an additional 17 cases which were not tested for HIV-1 status. Controls for the various studies were defined as persons without a previous history of tuberculosis and were frequency matched for age, sex and area of residence. A number of clinical correlates of tuberculosis were recorded for the The Gambia (A), Republic of Guinea and Guinea Bissau studies including haemoptysis, duration of cough, degree of sputum positivity, and the presence of cavitation and number of zones affected on chest radiograph. Cavitory tuberculosis status and number of zones affected was determined by radiological analysis by board-certified radiologists.

Informed written consent was obtained from patients or their parents or guardians. Ethical approval was provided by the joint Gambian Government/MRC Ethical Committee, Ministry of Public Health (MINSAP, Guinea-Bissau) and National Ethics Committee, Ministry of Health, Conakry, Republique de Guinee. Human experimentation guidelines of these ministries were followed. Ethical approval for the Malawi study protocol was obtained from the National Health Sciences Research Committee of Malawi and the London School of Hygiene and Tropical Medicine Ethics Committee.

### Genotyping techniques

DNA extraction from blood was performed using Nucleon II kits (Scotlab Bioscience, Buckingham, UK). DNA concentrations were determined using the PicoGreen™ kit (Invitrogen, Carlsbad, USA). Genotyping was performed utilizing the Sequenom™ system (Sequenom, San Diego, USA) which uses mass spectrometry (MALDI-TOF) to discriminate products by their absolute mass [36]. Primer extension was carried out utilizing a DNA primer adjacent to the SNP, and a specific reaction mix of polymerase, dNTPs and one ddNTPs. The extension products were then cleaned up to remove salts and 15nl were spotted onto a 384 SpectroCHIP (Sequenom). This chip was analysed by MALDI-TOF mass spectrometer and the alleles called by weight (in kilodaltons) of the extension products. The *CD209* -871A/G (rs735239) and -336A/G (rs4804803) SNP assays were designed using the SPECTRODESIGNER software (Sequenom). The primers for the *CD209* -871A/G were ACGTTGGATGCTCTGTCTGGGTCCTTTTAC, ACGTTGGATGACAGCAATGAAAAAGCAAAG, GCAAAGTACTAGTACATTTAATAAC and for *CD209* -336A/G were ACGTTGGATGTGTTACACCCCCTCCACTAG, ACGTTGGATGAAAGCAGGAAAGCCAGGAGG, CCCTCCACTAGGGCAAGGGT. The assays were in 384-well plates containing 10 ng of DNA in each well were amplified by a touch-down PCR using Titanium Taq polymerase (Clontech). The touch-down PCR cycling conditions were as follows: 95°C for 15 minutes; 94°C for 20 seconds; 65°C for 30 seconds; 72°C for 30 seconds; steps 2 to 4 repeated for 5 cycles; 94°C for 20 seconds; 58°C for 30 seconds; 72°C for 30 seconds; steps 5 to 7 repeated for 5 cycles; 94°C for 20 seconds; 53°C for 30 seconds; 72°C for 30 seconds; steps 8 to 10 repeated for 38 cycles; final extension at 72°C for 3 minutes.

### Statistical analysis

Statistical analysis of genotype associations and logistic regression was performed using the program SPSS v14.0. The genotypic and allelic tests of association utilized Pearson's chi-square test with the respective degrees of freedom. All statistics are two-tailed. If the unadjusted *P* value was significant then further analysis was carried out using a stepwise forward binary logistic regression correction to adjust for the potential confounding factors of HIV status, age, ethnicity, and sex. Mantel-Haenszel for the combined analysis was performed using SPSS by weighting cases by frequency and stratifying by population. The Gambian (B) study utilizes the Gambian (A) study controls for this Mantel-Haenszel analysis. The Breslow-Day and Tarone's tests for homogeneity of odds ratios were found to be non-significant indicating that the odds ratio for each study did not significantly deviate from the overall, combined odds ratio. All control genotype distributions were in Hardy-Weinberg equilibrium (*P*>0.20).

## Results

### CD209 and association with tuberculosis

The *CD209* -871A/G and -336A/G SNPs were initially genotyped in 329 tuberculosis cases and 327 control individuals from The Gambia (study group A) [33]. The *CD209* -871 SNP was found to be polymorphic with controls and cases both having a 2.3% allele frequency (allelic *P* = 0.98) consistent with the Barreiro result [30] and the HapMap project which found variation at this SNP to be very rare to absent in individuals of African descent. The allelic equivalence between cases and controls and the rarity of this SNP precluded genotyping in further populations. For the CD209 -336A/G SNP, however, an overall genotypic association with clinical tuberculosis was observed for this cohort (3x2χ^2^ = 7.79, *P* = 0.020) (**Table 1**). The -336G allele was significantly under-represented in tuberculosis cases (47.0%) when compared to healthy controls (54.0%) (*P* = 0.010, odds ratio of 0.75 (95%CI 0.61–0.94) (**Table 2**). Logistic regression correction for age, sex, ethnic group and HIV-1 status did not significantly affect this association. Subsequently an additional 347 tuberculosis cases from The Gambia were available for genotyping (study group B), revealing a similar tuberculosis case allele frequency (48.0%) and a significant association when analyzed against the study group A control group [34].

**Table 1:** *CD209* -336A/G SNP in Patients Enrolled in Tuberculosis Case Control Studies in Four Countries

Variant	The Gambia			Republic of Guinea		Guinea-Bissau		Malawi	
	Cases (A)	Cases (B)	Controls	Cases	Controls	Cases	Controls	Cases	Controls
	n = 329	n = 347	n = 327	n = 151	n = 180	n = 162	n = 141	n = 244	n = 295
AA	26.7	28.0	22.0	28.3	29.8	32.7	25.5	43.5	38.3
AG	52.6	48.1	48.0	45.6	46.4	39.5	48.2	42.6	50.5
GG	20.7	23.9	30.0	26.1	23.8	27.8	26.3	13.9	11.2
χ^2^	7.79	4.66	–	0.24	–	2.71	–	3.45	–
*P* value	**0.020**	0.097	–	0.886	–	0.258	–	0.178	–

Genotypic frequency and genotypic association (3×2 χ^2^, 2 degrees of freedom) for *CD209* -336A/G (rs4804803) from 2176 individuals enrolled in tuberculosis case control studies from The Gambia (A), Republic of Guinea and Guinea-Bissau (ref. 33), The Gambia (B) (ref. 34) and Malawi (ref. 35). Significance level of *P*<0.05 is indicated in bold.

**Table 2:** *CD209* -336A/G SNP Allelic Association in Tuberculosis Case Control Studies

Variant	The Gambia (A)	The Gambia (B)*	Republic of Guinea	Guinea-Bissau	Malawi	Overall (M-H)
G allele Controls	353 (54.0%)	353 (54.0%)	142 (47.0%)	142 (50.4%)	215 (36.4%)	–
G allele Cases	309 (47.0%)	333 (48.0%)	176 (48.9%)	154 (47.5%)	172 (35.2%)	–
Odds Ratio	0.75	0.79	1.08	0.89	0.95	0.86
95% CI	0.61–0.94	0.64–0.97	0.79–1.46	0.65–1.23	0.74–1.22	0.77–0.96
*P* value	**0.010**	**0.028**	0.632	0.488	0.684	**0.006**

The allelic *P* value for 2176 individuals enrolled in tuberculosis case control studies. The Gambia (A), Republic of Guinea and Guinea-Bissau (ref. 33), The Gambia (B) (ref. 34) and Malawi (ref. 35). *The Gambia (B) study utilizes The Gambia (A) controls. Significance levels of *P*<0.05 are indicated in bold.

Study groups from Republic of Guinea, Guinea-Bissau, and Malawi were then used in an attempt to replicate this finding [33], [37]. Each study group, with the exception of Republic of Guinea study, demonstrated the same trend towards a protective effect of the -336G allele. In total 2176 individuals were analyzed in this study and combining the results in a Mantel-Haenszel test predicted a protective effect of the -336G allele with an overall odds ratio of 0.86 (95%CI 0.77–0.96) and *P* = 0.006 (**Table 2**). This allelic model yielded a non-significant homogeneity of odds ratio between all the study groups, (Tarone's and Breslow-Day *P* = 0.32), suggesting that there was a good consistency between the combined odds ratio with the individual odds ratios for each study.

Considering that HIV-1 status could potentially confound this association we performed the Mantel-Haenszel test excluding all individuals known to be HIV positive, giving an odds ratio of 0.83 (95%CI 0.73–0.94) and *P* = 0.003 (**Table S1**). Although this exclusion of HIV-infected individuals reduces the total sample size of the study the conclusion remains unchanged.

### CD209 and clinical correlates of tuberculosis

**Figure 1:**
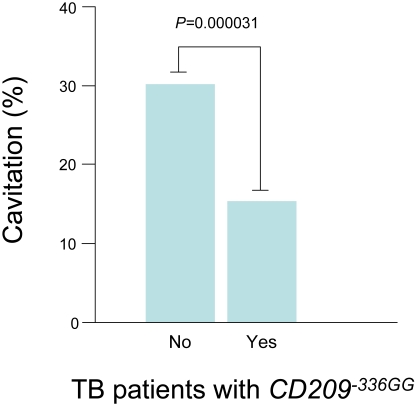
*CD209* -336GG and *M. tuberculosis*-induced lung cavitation. This figure describes the percent of individuals with the *CD209* -336GG genotype who presented with cavitory tuberculosis disease.

The *CD209* -336A/G SNP was further examined in the cases for association with clinical correlates of tuberculosis, with no significant difference between sex, HIV status, age, haemoptysis, number of zones affected on chest radiograph, duration of cough or degree of sputum positivity. However, a significant association was obtained between *CD209* -336GG genotype status and lung cavitation in tuberculosis patients. Patients homozygous for the *CD209* -336GG allele were associated with 2.4-fold protection against cavitory tuberculosis disease (n = 557, Pearson's 2×2 χ^2^ = 17.34, *P* = 0.00003, odds ratio = 0.42, 95%CI 0.27–0.65, *P*_Bonferroni corrected_ = 1.19×10^−4^)(**Figure 1** and **Table 3**).

**Table 3:** *CD209* -336A/G and Clinical Correlates of Tuberculosis

Clinical Correlates	Status	*CD209* -336A/G variant*		Total	*P* value	Odds Ratio	95% CI
		AA+AG	GG				
Sex	Male	573 (73.4%)	208 (26.6%)	781	0.293	0.871	0.673–1.127
	Female	389 (76.0%)	123 (24.0%)	512			
HIV	Negative	831 (73.8%)	295 (26.2%)	1126	0.255	1.306	0.823–2.073
	Positive	92 (78.6%)	25 (21.4%)	117			
Age Groups	0–19	100 (75.8%)	32 (24.2%)	132	0.996		
	20–29	363 (74.1%)	127 (25.9%)	490			
	30–39	264 (74.6%)	90 (25.4%)	354			
	40–49	145 (74.4%)	50 (25.6%)	195			
	50–99	90 (73.8%)	32 (26.2%)	122			
Haemoptysis	No	281 (77.2%)	83 (22.8%)	364	0.858	0.959	0.604–1.523
	Yes	113 (77.9%)	32 (22.2%)	145			
Cavitation	No	194 (69.8%)	84 (30.2%)	278	**0.0000313**	0.421	0.278–0.636
	Yes	236 (84.6%)	43 (15.4%)	279			
No. Zones affected	1–3	182 (77.1%)	54 (22.9%)	236	0.931	0.982	0.658–1.468
	4–6	247 (77.4%)	72 (22.6%)	319			
Duration of cough	0–8 weeks	271 (79.0%)	72 (21.0%)	343	0.259	1.285	0.831–1.989
	>8 weeks	123 (74.5%)	42 (25.5%)	165			
Degree of sputum positivity	+	12 (66.7%)	6 (33.3%)	18	0.604		
	++	162 (75.7%)	52 (24.3%)	214			
	+++	337 (76.8%)	102 (23.2%)	439			

* Combined West African tuberculosis case control data. Significance level of *P*<0.05 is indicated in bold.

## Discussion

There is increasing evidence to support an important role for DC-SIGN in the pathophysiology of a number of infectious diseases, including *M. tuberculosis* infection.

Functional evidence suggests that the -336G variant has lower basal expression of *CD209* when compared to the -336A ancestral allele [32]. Extrapolation of these *in vitro* experiments to *in vivo CD209* expression suggests that -336G individuals would have lower levels of DC-SIGN on DCs and alveolar macrophages when compared to -336A individuals. Barreiro and colleagues found an association with the -336A allele and protection against tuberculosis and suggest that higher levels of DC-SIGN may be beneficial in protection against tuberculosis disease.

**Figure 2:**
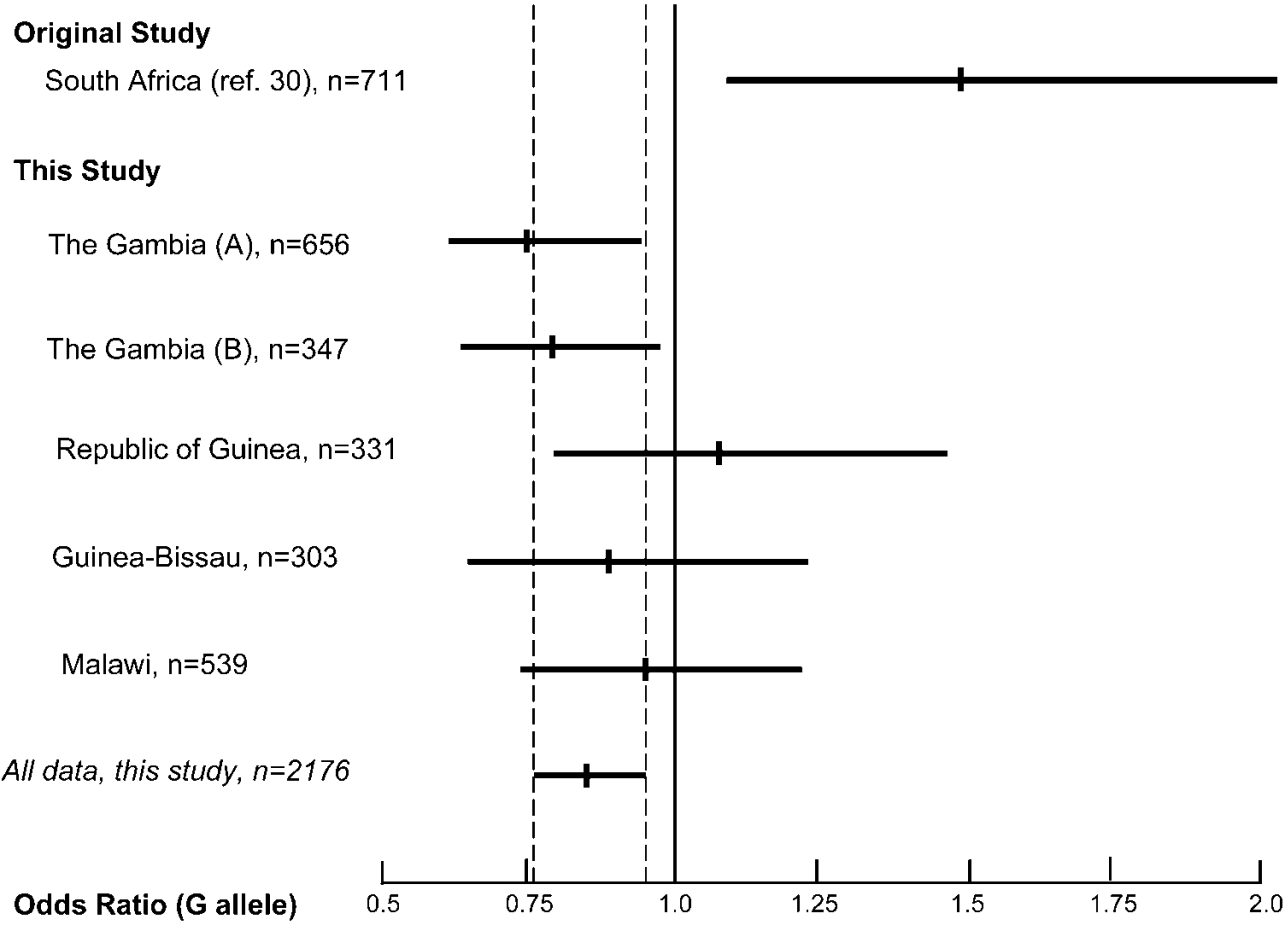
Odds ratio plot (with 95% confidence intervals) for *CD209* -336A/G. The odds ratio (with 95% confidence intervals) for *CD209* -336A/G for each of the studies are shown. Studies include the Barreiro study (ref. 30), The Gambia (A) (ref. 33), The Gambia (B) (ref. 34), Republic of Guinea (ref. 33), Guinea-Bissau (ref. 33) and Malawi (ref. 35). The central mark on each line indicates the odds ratio for each study and the line indicates that 95% confidence interval for each. The dashed line indicates the extent of the 95% confidence interval for the combined analysis of the populations analyzed for this study.

Here we report studies of the *CD209* -336A/G SNP in multiple tuberculosis case control groups based in The Gambia, Republic of Guinea, Guinea-Bissau, and Malawi. These studies, taken together, reveal a genetic association with the -336G allele and protection against tuberculosis. This protection was independent of HIV-1 status as assessed by logistic regression as well as removing all known HIV-1 cases from the analysis (**Table S1**). These results differ from the Barrerio study [30]. This discrepancy could reflect a chance finding in the original single study group, since the confidence interval for this population overlaps with those of our three smaller studies (**Figure 2**). Barreiro and colleagues studied a South African Coloured population known to have the -871 variant, which was absent in all of the sub-Saharan African populations previously genotyped and very rare (<2.5%) in this study. Indeed, it was the −871 variant which showed the most compelling evidence of association, with the −336 variant failing to reach even the nominal significance level of *P* = 0.05 after correcting for multiple testing. Due to the relative absence of the −871 variant in the African populations genotyped in this present study we could not ascertain the effect of this variant in susceptibility to tuberculosis and it is entirely possible that the −871/−336 haplotype drives differential *CD209* expression patterns but further work will be needed to confirm this.

Subgroup analysis suggested a significant association between *CD209* -336GG genotype status and protection against lung cavitation. The potential role of DC-SIGN in cavitory tuberculosis disease is novel and warrants further investigation. In this study we are not able to distinguish between cavitory tuberculosis caused by progression from primary infection, re-infection, or indeed by canonical post-primary reactivation disease. Cavitory tuberculosis in this setting defines a particularly severe manifestation of tuberculosis. Pulmonary cavitation in tuberculosis is known to be associated with the production of Th2 cytokines such as IL-4, which have in turn been demonstrated to result in higher surface expression levels of DC-SIGN in DCs [38]–[40]. This raises the interesting question of whether individuals homozygous for the -336G allele may, in part, be protected from expressing high levels of DC-SIGN and therefore less prone to the Th2 shift induced by ManLam binding to DC-SIGN [22], [29], [41], [42].

Maturation of DCs, defined by DC upregulation of co-stimulatory molecules such as CD80/CD86, can occur on exposure to TLR ligands. This process can however be inhibited by a variety of unknown mechanisms. One possible mechanism of inhibition of DC maturation is the binding of pattern recognition receptors to molecular patterns present on host cells [43]. This process, in theory, would cause DC anergy and a reduction in the pro-inflammatory response, even in the presence of pathogen-associated molecular patterns. Virulent *Mycobacterium spp.* have evolved mechanisms to more heavily decorate the cell wall lipoglycoconjugates with α(1→2) mannosylated termini which closely resemble the high mannose N-linked oligosaccharides of newly produced glycoproteins in eukaryotic cells [44]. Perhaps this molecular mimicry could be part of the mechanism by which virulent strains of *M. tuberculosis* modulate the immune response. If so, DC-SIGN is a prime target for this virulence strategy.

Overall, our results suggest that lower levels of DC-SIGN on immune subsets may be protective against tuberculosis in the sub-Saharan Africa setting. The findings implicate the involvement of DC-SIGN in the pathogenesis of clinical tuberculosis in general, and severe, cavitory tuberculosis in particular. This has wider implications since it provides further evidence that *M. tuberculosis* may use the DC-SIGN receptor to subvert the immune response.

## Supporting Information

Table S1 *CD209*-336A/G SNP Allelic Association in Tuberculosis Case Control Studies Excluding All HIV-1 Positive Cases and Controls. The allelic *P* value for HIV-1 negative individuals enrolled in tuberculosis case control studies. The Gambia (A), Republic of Guinea and Guinea-Bissau (ref. 33), The Gambia (B) (ref. 34) and Malawi (ref. 35). *The Gambia (B) study utilizes The Gambia (A) controls. Significance levels of *P*<0.05 are indicated in bold.
(0.06 MB DOC)

## References

[1] (2003). WHO annual report on global TB control–summary.. Wkly Epidemiol Rec.

[2] Frieden TR, Sterling TR, Munsiff SS, Watt CJ, Dye C (2003). Tuberculosis.. Lancet.

[3] Flynn JL, Chan J (2001). Immunology of tuberculosis.. Annu Rev Immunol.

[4] Cooke GS, Hill AV (2001). Genetics of susceptibility to human infectious disease.. Nat Rev Genet.

[5] Comstock GW (1978). Tuberculosis in twins: a re-analysis of the Prophit survey.. Am Rev Respir Dis.

[6] Stead WW, Senner JW, Reddick WT, Lofgren JP (1990). Racial differences in susceptibility to infection by Mycobacterium tuberculosis.. N Engl J Med.

[7] Doffinger R, Dupuis S, Picard C, Fieschi C, Feinberg J (2002). Inherited disorders of IL-12- and IFNgamma-mediated immunity: a molecular genetics update.. Mol Immunol.

[8] Soilleux EJ, Barten R, Trowsdale J (2000). DC-SIGN; a related gene, DC-SIGNR; and CD23 form a cluster on 19p13.. J Immunol.

[9] Tailleux L, Pham-Thi N, Bergeron-Lafaurie A, Herrmann JL, Charles P (2005). DC-SIGN induction in alveolar macrophages defines privileged target host cells for mycobacteria in patients with tuberculosis.. PLoS Med.

[10] Tailleux L, Schwartz O, Herrmann JL, Pivert E, Jackson M (2003). DC-SIGN is the major Mycobacterium tuberculosis receptor on human dendritic cells.. J Exp Med.

[11] Gordon S (2002). Pattern recognition receptors: doubling up for the innate immune response.. Cell.

[12] Geijtenbeek TB, Krooshoop DJ, Bleijs DA, van Vliet SJ, van Duijnhoven GC (2000). DC-SIGN-ICAM-2 interaction mediates dendritic cell trafficking.. Nat Immunol.

[13] Geijtenbeek TB, Torensma R, van Vliet SJ, van Duijnhoven GC, Adema GJ (2000). Identification of DC-SIGN, a novel dendritic cell-specific ICAM-3 receptor that supports primary immune responses.. Cell.

[14] Tailleux L, Maeda N, Nigou J, Gicquel B, Neyrolles O (2003). How is the phagocyte lectin keyboard played? Master class lesson by Mycobacterium tuberculosis.. Trends Microbiol.

[15] van Kooyk Y, Appelmelk B, Geijtenbeek TB (2003). A fatal attraction: Mycobacterium tuberculosis and HIV-1 target DC-SIGN to escape immune surveillance.. Trends Mol Med.

[16] Alvarez CP, Lasala F, Carrillo J, Muniz O, Corbi AL (2002). C-type lectins DC-SIGN and L-SIGN mediate cellular entry by Ebola virus in cis and in trans.. J Virol.

[17] Appelmelk BJ, van Die I, van Vliet SJ, Vandenbroucke-Grauls CM, Geijtenbeek TB (2003). Cutting edge: carbohydrate profiling identifies new pathogens that interact with dendritic cell-specific ICAM-3-grabbing nonintegrin on dendritic cells.. J Immunol.

[18] Barreiro LB, Quach H, Krahenbuhl J, Khaliq S, Mohyuddin A (2006). DC-SIGN interacts with Mycobacterium leprae but sequence variation in this lectin is not associated with leprosy in the Pakistani population.. Hum Immunol.

[19] Bergman MP, Engering A, Smits HH, van Vliet SJ, van Bodegraven AA (2004). Helicobacter pylori modulates the T helper cell 1/T helper cell 2 balance through phase-variable interaction between lipopolysaccharide and DC-SIGN.. J Exp Med.

[20] Colmenares M, Puig-Kroger A, Pello OM, Corbi AL, Rivas L (2002). Dendritic cell (DC)-specific intercellular adhesion molecule 3 (ICAM-3)-grabbing nonintegrin (DC-SIGN, CD209), a C-type surface lectin in human DCs, is a receptor for Leishmania amastigotes.. J Biol Chem.

[21] Geijtenbeek TB, Kwon DS, Torensma R, van Vliet SJ, van Duijnhoven GC (2000). DC-SIGN, a dendritic cell-specific HIV-1-binding protein that enhances trans-infection of T cells.. Cell.

[22] Geijtenbeek TB, Van Vliet SJ, Koppel EA, Sanchez-Hernandez M, Vandenbroucke-Grauls CM (2003). Mycobacteria target DC-SIGN to suppress dendritic cell function.. J Exp Med.

[23] Halary F, Amara A, Lortat-Jacob H, Messerle M, Delaunay T (2002). Human cytomegalovirus binding to DC-SIGN is required for dendritic cell infection and target cell trans-infection.. Immunity.

[24] Koppel EA, Ludwig IS, Hernandez MS, Lowary TL, Gadikota RR (2004). Identification of the mycobacterial carbohydrate structure that binds the C-type lectins DC-SIGN, L-SIGN and SIGNR1.. Immunobiology.

[25] Lozach PY, Amara A, Bartosch B, Virelizier JL, Arenzana-Seisdedos F (2004). C-type lectins L-SIGN and DC-SIGN capture and transmit infectious hepatitis C virus pseudotype particles.. J Biol Chem.

[26] Pohlmann S, Soilleux EJ, Baribaud F, Leslie GJ, Morris LS (2001). DC-SIGNR, a DC-SIGN homologue expressed in endothelial cells, binds to human and simian immunodeficiency viruses and activates infection in trans.. Proc Natl Acad Sci U S A.

[27] Pohlmann S, Zhang J, Baribaud F, Chen Z, Leslie GJ (2003). Hepatitis C virus glycoproteins interact with DC-SIGN and DC-SIGNR.. J Virol.

[28] Tassaneetrithep B, Burgess TH, Granelli-Piperno A, Trumpfheller C, Finke J (2003). DC-SIGN (CD209) mediates dengue virus infection of human dendritic cells.. J Exp Med.

[29] van Kooyk Y, Geijtenbeek TB (2003). DC-SIGN: escape mechanism for pathogens.. Nat Rev Immunol.

[30] Barreiro LB, Neyrolles O, Babb CL, Tailleux L, Quach H (2006). Promoter variation in the DC-SIGN-encoding gene CD209 is associated with tuberculosis.. PLoS Med.

[31] Martin MP, Lederman MM, Hutcheson HB, Goedert JJ, Nelson GW (2004). Association of DC-SIGN promoter polymorphism with increased risk for parenteral, but not mucosal, acquisition of human immunodeficiency virus type 1 infection.. J Virol.

[32] Sakuntabhai A, Turbpaiboon C, Casademont I, Chuansumrit A, Lowhnoo T (2005). A variant in the CD209 promoter is associated with severity of dengue disease.. Nat Genet.

[33] Bennett S, Lienhardt C, Bah-Sow O, Gustafson P, Manneh K (2002). Investigation of environmental and host-related risk factors for tuberculosis in Africa. II. Investigation of host genetic factors.. Am J Epidemiol.

[34] Bellamy R, Ruwende C, Corrah T, McAdam KP, Whittle HC (1998). Variations in the NRAMP1 gene and susceptibility to tuberculosis in West Africans.. N Engl J Med.

[35] Fitness J, Floyd S, Warndorff DK, Sichali L, Malema S (2004). Large-scale candidate gene study of tuberculosis susceptibility in the Karonga district of northern Malawi.. Am J Trop Med Hyg.

[36] Storm N, Darnhofer-Patel B, van den Boom D, Rodi CP (2003). MALDI-TOF mass spectrometry-based SNP genotyping.. Methods Mol Biol.

[37] Lienhardt C, Bennett S, Del Prete G, Bah-Sow O, Newport M (2002). Investigation of environmental and host-related risk factors for tuberculosis in Africa. I. Methodological aspects of a combined design.. Am J Epidemiol.

[38] Condos R, Rom WN, Liu YM, Schluger NW (1998). Local immune responses correlate with presentation and outcome in tuberculosis.. Am J Respir Crit Care Med.

[39] Mazzarella G, Bianco A, Perna F, D'Auria D, Grella E (2003). T lymphocyte phenotypic profile in lung segments affected by cavitary and non-cavitary tuberculosis.. Clin Exp Immunol.

[40] van Crevel R, Karyadi E, Preyers F, Leenders M, Kullberg BJ (2000). Increased production of interleukin 4 by CD4+ and CD8+ T cells from patients with tuberculosis is related to the presence of pulmonary cavities.. J Infect Dis.

[41] Soilleux EJ, Morris LS, Leslie G, Chehimi J, Luo Q (2002). Constitutive and induced expression of DC-SIGN on dendritic cell and macrophage subpopulations in situ and in vitro.. J Leukoc Biol.

[42] Soilleux EJ, Sarno EN, Hernandez MO, Moseley E, Horsley J (2006). DC-SIGN association with the Th2 environment of lepromatous lesions: cause or effect?. J Pathol.

[43] van Kooyk Y, Engering A, Lekkerkerker AN, Ludwig IS, Geijtenbeek TB (2004). Pathogens use carbohydrates to escape immunity induced by dendritic cells.. Curr Opin Immunol.

[44] Torrelles JB, Azad AK, Schlesinger LS (2006). Fine discrimination in the recognition of individual species of phosphatidyl-myo-inositol mannosides from Mycobacterium tuberculosis by C-type lectin pattern recognition receptors.. J Immunol.

