# Supplementary material for: CD209 Genetic Polymorphism and Tuberculosis Disease
Source: PLoS One. 2008 Jan 2;3(1):e1388. doi: 10.1371/journal.pone.0001388 (PMC2148105; doi:10.1371/journal.pone.0001388)
Supplement: Table S1 — CD209-336A/G SNP Allelic Association in Tuberculosis Case Control Studies Excluding All HIV-1 Positive Cases and Controls. The allelic P value for HIV-1 negative individuals enrolled in tuberculosis case control studies. The Gambia (A), Republic of Guinea and Guinea-Bissau (ref. 33), The Gambia (B) (ref. 34) and Malawi (ref. 35). *The Gambia (B) study utilizes The Gambia (A) controls. Significance levels of P<0.05 are indicated in bold. (0.06 MB DOC) [file pone.0001388.s001.doc]

| **Table S1.** *CD209* -336A/G SNP Allelic Association in Tuberculosis Case Control Studies Excluding All HIV-1 Positive Cases and Controls | | | | | | |
| --- | --- | --- | --- | --- | --- | --- |
|  | | | | | | |
| **Variant** | **The Gambia (A)** | **The Gambia (B)*** | **Republic of Guinea** | **Guinea-Bissau** | **Malawi** | **Overall (M-H)** |
| G allele Controls | 330 (54.3%) | 330 (54.3%) | 129 (45.7%) | 101 (54.3%) | 196 (36.8%) | -- |
| G allele Cases | 289 (47.2%) | 333 (48.0%) | 156 (49.4%) | 116 (46.8%) | 64 (34.8%) | -- |
| Odds Ratio | 0.75 | 0.78 | 1.16 | 0.74 | 0.91 | 0.83 |
| 95% CI | 0.60-0.94 | 0.63-0.97 | 0.84-1.60 | 0.512-1.08 | 0.64-1.30 | 0.73-0.94 |
| *P* value | **0.014** | **0.023** | 0.376 | 0.121 | 0.617 | **0.003** |

The allelic *P* value for HIV-1 negative individuals enrolled in tuberculosis case control studies. The Gambia (A), Republic of Guinea and Guinea-Bissau (ref. 33), The Gambia (B) (ref. 34) and Malawi (ref. 35). *The Gambia (B) study utilizes The Gambia (A) controls. Significance levels of *P* < 0.05 are indicated in bold.
